# Exogenous Nitric Oxide Delays Plant Regeneration from Protoplast and Protonema Development in *Physcomitrella* *patens*

**DOI:** 10.3390/plants9101380

**Published:** 2020-10-16

**Authors:** Daniela Cervantes-Pérez, Angélica Ortega-García, Rigoberto Medina-Andrés, Ramón Alberto Batista-García, Verónica Lira-Ruan

**Affiliations:** Centro de Investigación en Dinámica Celular, Instituto de Investigación en Ciencias Básicas y Aplicadas, Universidad Autónoma del Estado de Morelos. Av. Universidad 1001. Col Chamilpa. Cuernavaca, Morelos 62210, Mexico; danielacp31@gmail.com (D.C.-P.); oga_ff@uaem.mx (A.O.-G.); spidermunro@hotmail.com (R.M.-A.); rabg@uaem.mx (R.A.B.-G.)

**Keywords:** cell growth, nitric oxide, *Physcomitrellapatens*, protoplast

## Abstract

Nitric oxide (NO) has been recognized as a major player in the regulation of plant physiology and development. NO regulates cell cycle progression and cell elongation in flowering plants and green algae, although the information about NO function in non-vascular plants is scarce. Here, we analyze the effect of exogenous NO on *Physcomitrella patens* protonema growth. We find that increasing concentrations of the NO donor sodium nitroprusside (SNP) inhibit protonema relative growth rate and cell length. To further comprehend the effect of NO on moss development, we analyze the effect of SNP 5 and 10 µM on protoplast regeneration and, furthermore, protonema formation compared with untreated plants (control). Isolated protoplasts were left to regenerate for 24 h before starting the SNP treatments that lasted five days. The results show that SNP restrains the protoplast regeneration process and the formation of new protonema cells. When SNP treatments started five days after protoplast isolation, a decrease in cell number per protonema filament was observed, indicating an inhibition of cell cycle progression. Our results show that in non-vascular plants, NO negatively regulates plant regeneration, cell cycle and cell elongation.

## 1. Introduction

Nitric oxide (NO) is a versatile molecule that influences plant physiology and development from germination to senescence and in response to biotic and abiotic stresses [1,2] In land plants, NO is enzymatically generated by nitric oxide synthase-like activity (reviewed by Astier et al [3] and nitrate reductase (NR) [4]. Non-enzymatic production of NO is promoted by polyamines [5], electron transport in mitochondria [6] and peroxysomes [7]. The message of NO is transmitted by the posttranslational modification of Cys residues (S-nitrosylation), Tyr residues (nitration) and metal nitrosylation of proteins, thus directly affecting protein function and gene expression [8]. Moreover, NO cross-talk with phytohormones such as auxin, cytokinins and abscisic acid regulates plant development and environmental responses [9].

A great volume of knowledge about NO roles in plant physiology has been obtained by altering NO levels using NO donors, like sodium nitroprusside (SNP), S-nitroso-*N*-acetylpenicillamine (SNAP) and S-nitrosoglutathione (GSNO) and the NO scavenger 2-(4Carboxyphenyl)-4,4,5,5-tetramethylimidazoline-1-oxyl-3-oxide (cPTIO). In 1997, SNP was used to demonstrate the role of NO in the elongation of maize hypocotyl segments [10]. Treatment with SNP and cPTIO in tomato and cucumber roots showed the role of NO in the formation of new lateral and adventitious roots, respectively [11,12]. In tomato and *Arabidopsis thaliana* roots, exogenous NO derived from SNP restrained primary root growth by altering the meristematic activity and inhibiting cell elongation [13,14]. 

The analysis of the role of NO in the cell cycle and elongation has extended to different cell types and plants. In *A. thaliana* and lettuce, NO increased root hair formation [15]. In green algae, NO also affects cell growth; the addition of the NO donor SNAP suppressed cell growth in *Micrasterias denticulata* [16]. An increase in endogenous NO levels at the beginning of the lag phase indicates its role in promoting active cell growth in the unicellular algae *Chlorella vulgaris* and *Chlamydomonas* sp. [17]. In *Chlamydomonas* sp., NO has a role in cell cycle progression [18]. Regardless of the increasing evidence of NO function in growth from simple unicellular photosynthetic eukaryotes to complex seed plants, there is almost no information regarding NO in non-vascular plants. *Physcomitrella patens* is a useful model to understand complex plant physiology and developmental processes such as hormone response, stem cell formation and maintenance, and plant–pathogen interactions [19,20,21,22] We previously demonstrated that endogenous NO in *P. patens* is produced mainly by NR activity [23]. The pharmacological inactivation of NR resulted in the depletion of NO and a slight decrease in plant relative growth rate, suggesting a role of NO in *P. patens* cellular growth. Here, we show that increasing concentrations of the NO donor SNP decreases the protonema relative growth rate and cell elongation in a dose-dependent manner. The application of SNP on regenerating protoplasts delays plant regeneration and further chloronema development, indicating that in *P. patens*, NO affects both the cell cycle and cell elongation. 

## 2. Materials and Methods

### 2.1. Plant Material and Growth Conditions

The moss *Physcomitrella patens* (Hewd) B.S.G. was cultivated on solid Knop media [24] covered with sterile cellophane discs. Plants were cultivated in a growth chamber at 21 °C with a 16/8 h light/dark period at 30 µmol m^−2^ s^−1^. To get chloronema-enriched cultures for protoplast isolation, 15-day-old plants cultivated as described were homogenized using a T18 digital ultraturrax homogenizer (IKA), and homogenized tissue was cultivated as described for 10 days and the homogenization process was repeated twice. The tissue obtained after three homogenization steps was used to isolate protoplasts.

### 2.2. Exogenous NO Treatments

To analyze the effect of exogenous NO on *P. patens* protonema growth, pieces of protonema were taken from plants cultivated for seven days. The protonema pieces were transferred to fresh Knop medium (untreated control) or to the same medium supplemented with different concentrations of NO donor, sodium nitroprusside (SNP), with or without the NO scavenger 2-(4-carboxyphenyl)-4,4,5,5-tetramethyl imidazoline-1-oxyde-oxyl-3-oxyde (cPTIO). The plants were grown for seven days under the treatments, changed every day to fresh medium with fresh treatments. The Petri dishes with the protonema cultures were photographed at the beginning and end of the experiment (days 0 and 7) with a Nikon Coolpix 5000 camera. 

### 2.3. Protoplast Isolation and NO Treatment

Protoplasts were isolated from chloronema-enriched cultures grown in solid Knop media at 23 °C with continuous light 30 µmol m^−2^ s^−1^. Protoplasts were isolated according to a known protocol [25]. The tissue was treated with driselase 2% in mannitol 9% for 1 h. Recovered protoplasts were washed twice with mannitol 9%. The protoplasts were suspended in Knop/mannitol 6% to have 60,000 protoplasts in cellophane discs covering each Petri dish (50 mm). After isolation, the cultures were placed back in the growth chamber. To study the NO effect on plant regeneration, the treatments with SNP and cPTIO started at day three after protoplast isolation and lasted for five days. To study the effect of NO on further plant development, the treatments with SNP and cPTIO started at day five after protoplast isolation and lasted for five more days. In all cases, the plants were transferred every day to fresh medium.

### 2.4. Plant Growth and Development Estimation

The areas covered by each plant at day zero and day seven were measured using ImageJ software [26]. The relative growth rate was calculated using the following formula: (*ln a_f_ − ln a*_0_*)t*^−1^ [27] where *a_f_* and *a*_0_ are the areas occupied by the plant on the final day of growth (day 7) and at the beginning of the experiment (day 0) and *t* is the duration of growth in days equal to 7 days. The length of the caulonema and chloronema cells was measured in samples of the newly formed protonema obtained at the end of the experiment. The protoplast regeneration and protonema development were analyzed every day for 100 plants from each treatment. All observations were performed with an inverted microscope (Nikon EclipseTi) equipped with a Nikon D7000 camera.

Three independent experiments were performed. ANOVA tests were applied to determine significant differences between the different cases. Firstly, two tests were used to demonstrate in the experimental data (i) homogeneity of variance and (ii) normal distribution: the Hartley–Cochran–Bartlett test and Kolmogorov–Smirnov test, respectively. Later, ANOVAs were conducted to demonstrate the similarities or differences between the data. Finally, a post hoc analysis (Tukey’s HSD) that defines the order of the differences found in the ANOVAs was performed. All statistical calculations were performed in STATISTICA (last version for computers).

## 3. Results and Discussion

### 3.1. NO Donor Decreases Relative Growth of P. patens

**Figure 1:**
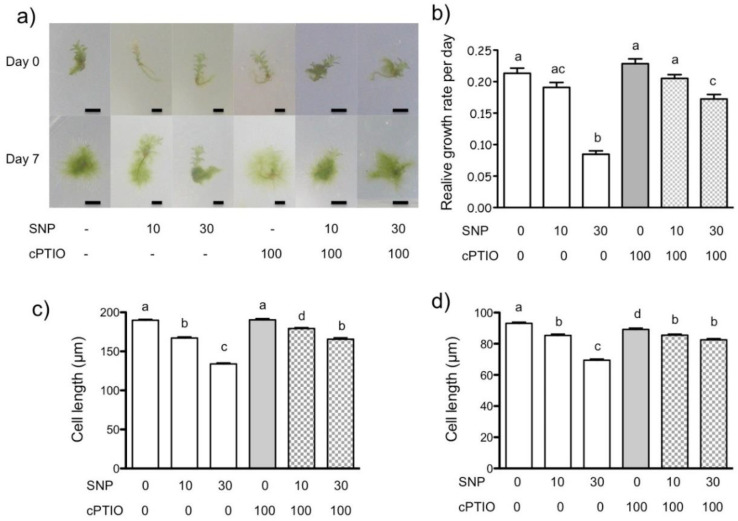
Effect of nitric oxide (NO) donors in *P. patens* growth. NO donor-dependent growth inhibition is reversed by a specific NO scavenger. (**a**) Comparison of plant area at the beginning (day 0) and the end (day 7) of treatment with the NO donor sodium nitroprusside (SNP) and/or NO scavenger 2-(4-carboxyphenyl)-4,4,5,5-tetramethylimidazoline-1-oxyde-oxyl-3-oxyde (cPTIO) at different concentrations in µM. (**b**) Comparison of the relative growth rate of *P. patens* protonema under NO donor and scavenger treatments. Final cell lengths of caulonema (**c**) and chloronema (**d**) cells. One representative experiment is shown in (**a**); scale bar = 3 mm. Data shown in (**b**) are from four independent experiments (*n* = 78), data shown in (**c**,**d**) are from four independent experiments (*n* = 286) and were analyzed by one-way ANOVA and Tukey’s multiple comparison test. Different letters indicate statistical differences among treatments at *p* < 0.05.

To analyze the NO effect on *P. patens* protonema growth, we treated seven-day-old plants with the NO donor SNP at 10 and 30 µM for seven days. Plants at the beginning and end of the experiment were compared. Plants appeared to be smaller as SNP concentration increased and the simultaneous addition of the NO scavenger cPTIO reduced that difference (Figure 1a). The quantitative analysis showed that only SNP 30 µM reduced the relative growth rate by more than a half compared to the control, and that growth reduction was partially reversed by simultaneous addition of cPTIO 100 µM, indicating that the NO derived from SNP was inhibiting plant growth (Figure 1b).

We also analyzed the effect of NO donor on caulonema and chloronema cell length (Figure 1c,d, respectively). In this case, both SNP concentrations reduced cell length in a dose-dependent manner. The caulonema and chloronema cells treated with SNP 10 µM were 9 and 7% shorter than untreated control plants, respectively. When treated with 30 µM SNP, they were 28 and 24% shorter that control plants, respectively. The simultaneous treatment with cPTIO partially relieved the effect of SNP. These experiments demonstrated that exogenous NO restrains *P. patens* growth. This effect is common to green algae and flowering plants. In the unicellular algae *Micrasterias denticulate*, two NO donors, SNP and SNAP, suppressed cell growth [16]. In *A. thaliana* roots treated with SNP, root growth was inhibited by reducing cortex cell length [14] and arresting the cell cycle in meristematic cells [13]. In *P. patens*, the observed decrease in cell length was less than the decrease in relative plant growth, suggesting that NO may also affect the cell cycle as in *A. thaliana* roots. To better understand the effect of NO in the reduction of *P. patens* growth and development, we analyzed the effect of SNP on plant regeneration and early development from protoplasts.

### 3.2. NO Donor Delays the Regeneration Process and Chloronema Initiation

**Figure 2:**
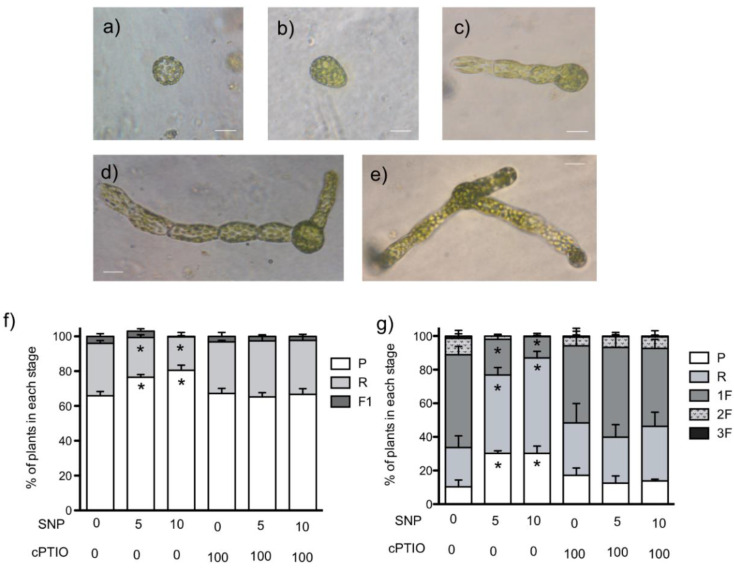
Effect of NO donors in protoplast regeneration and protonema initiation. Representative images of development stages observed during protoplast regeneration process: (**a**) protoplast; (**b**) regeneration started, (**c**) regenerated plant with one chloronema filament, (**d**) plant with two chloronema filaments and (**e**) plant with three chloronema filaments. Isolated protoplasts were allowed to initiate regeneration for 24 h before starting treatments with NO donor sodium nitroprusside (SNP) and/or scavenger 2-(4-carboxyphenyl)-4,4,5,5-tetramethylimidazoline-1-oxyde-oxyl-3-oxyde (cPTIO) at different concentrations in µM. Treatments lasted five days. (**f**) Percentage of plants in each development state at 3 days after isolation (DAI) and 2 days under treatment. (**g**) Percentage of plants in each development state at 7 DAI and 5 days under treatment. Scale bar = 30 µm (**a**,**b**); 50 µm (**c**–**e**). Three independent experiments were analyzed by one-way ANOVA and Tukey´s multiple comparison test. Asterisks indicate statistical differences with control at *p* < 0.05.

The formation of new plants from protoplasts is a useful tool to study plant regeneration in mosses. Thus, we isolated protoplasts and left them in the growth chamber to regenerate the cell wall for 24 h. Then they were treated with SNP and cPTIO for five days. Figure 2 shows the different stages of plant development we found. Round cells (Figure 2a) were considered to be protoplasts that had not started cell elongation (P), cells with an ovoid form (Figure 2b) were considered as regenerating plants (R), and plants with new chloronema (Figure 2c–e) were classified according to the number of filaments they formed (1F, 2F or 3F). The first experiment was performed using SNP 10 and 30 µM but after six days of treatment with SNP 30 µM, all protoplasts remained in the P or R stage, while in the control (without SNP or cPTIO), 70% of protoplasts had already regenerated in plants with one or more chloronema filaments (data not shown), demonstrating that protoplasts are much more sensitive to SNP than developed plants. Thus, we reduced the SNP concentration to 5 and 10 µM for further experiments. Right before starting the treatments with NO donor, it was observed that in all cultures, around 6% of the cells were in R stage and 94% were still in P stage. The progress of plant development was recorded every day for five days. Graphs for three and seven days after isolation (DAI) are shown in Figure 2.

Cell wall formation in *P. patens* starts one hour after protoplast isolation and the progress of it, revealed by the formation of asymmetric cells (R cells in this work) preparing for polar growth, can be detected as early as 12 h after isolation and as late as 50 h after isolation [28,29,30]. In our hands, this process was a little delayed; at 3 DAI in control conditions (Figure 2f), only 35% of plants were in the regeneration stage or had one emerged filament (1F), this delay could be the result of media composition; in previous works, the regeneration Knop media included sucrose and ammonium tartrate, whereas we used simple Knop media. Interestingly, the SNP treatments aggravated the development retardation; in the SNP 10 µM treatment, 80% of cells remained in the P stage. However, plant development continued and the NO effect was more evident in plants 7 DAI (Figure 2g). More than 60% of control plants developed one or more filaments, while SNP halted development in a dose-dependent manner, and majority of plants remained in the R stage and only 21% of the SNP 5 µM plants and 12% of SNP 10 µM plants formed one filament. The differences were reverted when the NO scavenger cPTIO was added to the media, indicating that the effect is due to NO. In control and cPTIO-treated plants, a small percentage of plants developed two filaments, but none of the SNP-treated plants did. These results suggest that NO acts to delay plant regeneration from protoplasts and the start of protonema formation. 

Protoplast regeneration and the following protonema growth by tip growth are complex processes influenced by several cues and plant hormones. Moss growth and development are regulated by several physical (e.g., light) and chemical cues, like nutrients and hormones [28,31]. It has been demonstrated that exogenous auxin (1 µM) retarded the *P. patens* protoplast division rate; moreover, blocking auxin extrusion with NPA (naphylphthalamic acid) totally abolished the regeneration process [32]. Here, we demonstrated that NO impairs cell regeneration and protonema formation, suggesting that in *P. patens*, the cross-talk between NO and auxin could function as in flowering plants, where it has been demonstrated that NO enhances auxin promotion of cell division in alfalfa protoplasts [33].

### 3.3. NO Donors also Inhibited Cell Cycle in Regenerated Plants

**Figure 3:**
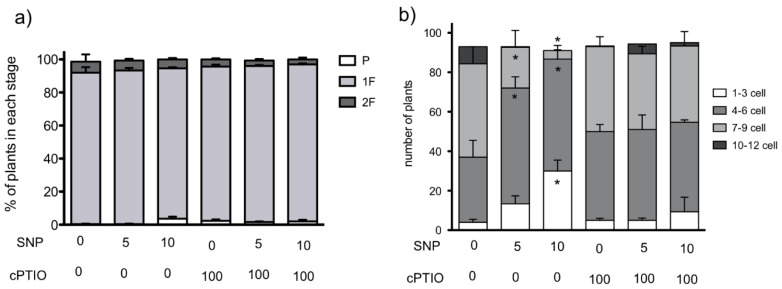
Effect of NO donors in young plant development. Isolated protoplasts were allowed to develop into plants for four days before starting the treatments with NO donor sodium nitroprusside (SNP) and/or scavenger 2-(4-carboxyphenyl)-4,4,5,5-tetramethylimidazoline-1-oxyde-oxyl-3-oxyde (cPTIO) at different concentrations in µM. Treatments lasted five days. (**a**) Percentage of plants in each development state at 10 days after isolation (DAI) and five days under treatment. (**b**) The number of cells in the plants in one filament (1F) stage in (**a**) were counted and compared between treatments. Four groups were formed: filaments with 1 to 3, 4 to 6, 7 to 9 and 10 to 12 cells/filament. Three independent experiments were analyzed by one-way ANOVA and Tukey´s multiple comparison test. Asterisks indicate statistical differences with control at *p* < 0.05.

In order to better evaluate the effect of NO donor in protonema formation and development, we allowed protoplasts to regenerate for four DAI, treatments with NO donor and scavenger started on day five after isolation and continued five more days. The analysis at six DAI (24 h of NO treatment) showed that approximately 70% of plants had developed one filament in all conditions (data not shown), indicating that at the beginning of the experiment, all plants were at the same developmental stage. These data confirm our previous observation that SNP delays polar growth set, as plants developed from protoplasts that were treated with SNP early in development (24 h after isolation) had only 12–21% of plants with 1F at 7 DAI (Figure 2g). After 5 days on the SNP treatment, the majority of plants had one filament in all treatments (Figure 3a). However, the number of cells in each filament changed when SNP was added; in control plants, the majority of filaments were seven to nine cells long (50 plants) and nine plants had more than 10 cells. In contrast, for SNP 5 µM, only 22 plants had seven to nine cells per filament and the majority of filaments were four to six cells long. In plants treated with SNP 10 µM, the majority of plants had filaments with four to six cells and, notably, the number of plants with filaments with one to three cells increased. These differences were reverted with cPTIO treatments, indicating that NO is responsible for this effect on cell cycle. The delay in filament formation and the decrease in filament cell number suggest that NO also impairs tip growth. 

As far as we know, there are no other studies on the effect of NO on tip growth in mosses, but it has been analyzed in pollen tube growth. Protoplast regeneration and protonema development are different from pollen tubes because, in addition to polarized tip growth, they must activate the cell cycle to produce a new organism. However, those results can shed some light on the analysis of our results. In *Lilium longiflorum*, the addition of NO donors inhibited tube growth and, moreover, the focused increase of NO near the tip arrested growth and promoted further re-orientation of the growth direction [34] and, in *Arabidopsis*, NO is needed for correct pollen tube growth and orientation towards the ovule [35]. Inhibition of pollen germination and elongation caused by NO donors was also reported in cucumber and *Pawlonia tomentosa* pollen [36,37]. During the germination of olive pollen grains, NO is produced along the pollen tube; the addition of exogenous NO reduced both germination and pollen tube growth rates [38]. Interestingly, the opposite effect was reported in *Pinus bungeana* where the treatment with SNAP increased the pollen tube germination and elongation rate [39]. The observed difference is attributable to the low pollen growth rate of *Pinus* compared to angiosperms [37,39]. Recently, Benko et al. [40] demonstrated in tobacco that the NO effect on pollen tube growth may be influenced by the balance between NO and ROS, adding complexity to the system. We previously showed endogenous NO in *P. patens* caulonemal cells [23], but detailed analysis of NO localization in the cell tip is needed in order to better understand NO’s role in cell elongation.

In summary, our work demonstrated that, in the basal lineage of land plants, exogenous NO negatively regulates the regeneration of protoplasts and the emergence of new protonema, apparently delaying cell division. The comparison of our results with other plant systems suggests that NO signaling may be common to all land plants, from bryophytes to angiosperms. Further studies are required to analyze the possible relations of NO and hormones during plant regeneration and tip growth in *P. patens*.

## References

[1] Besson-Bard A., Pugin A., Wendehenne D. (2008). New Insights into Nitric Oxide Signaling in Plants. Annu. Rev. Plant Biol..

[2] Moreau M., Lindermayr C., Durner J., Klessig D.F. (2010). NO synthesis and signaling in plants—Where do we stand?. Physiol. Plant..

[3] Astier J., Gross I., Durner J. (2017). Nitric oxide production in plants: An update. J. Exp. Bot..

[4] Yamasaki H., Sakihama Y. (2000). Simultaneous production of nitric oxide and peroxynitrite by plant nitrate reductase: In vitro evidence for the NR-dependent formation of active nitrogen species. FEBS Lett..

[5] Tun N., Santa-Catarina C., Begum T., Silveira V., Handro W., Floh E.I.S., Scherer G.F.E. (2006). Polyamines Induce Rapid Biosynthesis of Nitric Oxide (NO) in *Arabidopsis thaliana* Seedlings. Plant Cell Physiol..

[6] Gupta K.J., Kaiser W.M. (2010). Production and Scavenging of Nitric Oxide by Barley Root Mitochondria. Plant Cell Physiol..

[7] Corpas F.J., Barroso J.B., del Río L.A. (2001). Peroxisomes as a source of reactive oxygen species and nitric oxide signal molecules in plant cells. Trends Plant Sci..

[8] Baudouin E. (2010). The language of nitric oxide signalling. Plant Biol..

[9] Sanz L., Albertos P., Mateos I., Sánchez-Vicente I., Lechón T., Fernández-Marcos M., Lorenzo O. (2015). Nitric oxide (NO) and phytohormones crosstalk during early plant development. J. Exp. Bot..

[10] Gouvêa C., Souza J., Magalhães A., Martins I. (1997). NO—Releasing substances that induce growth elongation in maize root segments. Plant Growth Regul..

[11] Pagnussat G.C., Simontacchi M., Puntarulo S., LaMattina L. (2002). Nitric Oxide Is Required for Root Organogenesis. Plant Physiol..

[12] Correa-Aragunde N., Graziano M., Elamattina L. (2004). Nitric oxide plays a central role in determining lateral root development in tomato. Planta.

[13] Bai S., Li M., Yao T., Wang H., Zhang Y., Xiao L., Wang J.-Z., Zhang Z., Hu Y., Liu W. (2012). Nitric oxide restrain root growth by DNA damage induced cell cycle arrest in *Arabidopsis thaliana*. Nitric Oxide.

[14] Lira-Ruan V., Napsucialy S.N., Dubrovsky J.G. (2013). Heuristic aspect of the lateral root initiation index: A case study of the role of nitric oxide in root branching1. Appl. Plant Sci..

[15] Lombardo M.C., Graziano M., Polacco J.C., Elamattina L. (2006). Nitric Oxide Functions as a Positive Regulator of Root Hair Development. Plant Signal. Behav..

[16] Lehner C., Kerschbaum H.H., Lütz-Meindl U. (2009). Nitric oxide suppresses growth and development in the unicellular green alga *Micrasterias denticulata*. J. Plant Physiol..

[17] Estevez M.S., Puntarulo S. (2005). Nitric oxide generation upon growth of Antarctic *Chlorella* sp. cells. Physiol. Plant..

[18] Pokora W., Aksmann A., Baścik-Remisiewicz A., Dettlaff-Pokora A., Rykaczewski M., Gappa M., Tukaj Z. (2017). Changes in nitric oxide/hydrogen peroxide content and cell cycle progression: Study with synchronized cultures of green alga *Chlamydomonas reinhardtii*. J. Plant Physiol..

[19] Thelander M., Landberg K., Sundberg E. (2017). Auxin-mediated developmental control in the moss *Physcomitrella patens*. J. Exp. Bot..

[20] Kofuji R., Hasebe M. (2014). Eight types of stem cells in the life cycle of the moss *Physcomitrella patens*. Curr. Opin. Plant Biol..

[21] Ponce De León I., Schmelz E.A., Gaggero C., Castro A., Álvarez A., Montesano M. (2012). Physcomitrella patens activates reinforcement of the cell wall, programmed cell death and accumulation of evolutionary conserved defence signals, such as salicylic acid and 12-oxo-phytodienoic acid, but not jasmonic acid, upon Botrytis cinerea infection. Mol. Plant Pathol..

[22] Rensing S.A., Goffinet B., Meyberg R., Wu S.-Z., Bezanilla M. (2020). The Moss *Physcomitrium (Physcomitrella) patens*: A Model Organism for Non-Seed Plants. Plant Cell.

[23] Medina-Andrés R., Solano-Peralta A., Saucedo-Vázquez J.P., Napsucialy-Mendivil S., Pimentel-Cabrera J.A., Sosa-Torres M.E., Dubrovsky J.G., Lira-Ruan V. (2015). The Nitric Oxide Production in the Moss *Physcomitrella patens* is Mediated by Nitrate Reductase. PLoS ONE.

[24] Ashton N.W., Cove D.J. (1977). The isolation and preliminary characterisation of auxotrophic and analogue resistant mutants of the moss, *Physcomitrella patens*. Mol. Genet. Genom..

[25] Grimsley N.H., Ashton N.W., Cove D.J. (1977). The production of somatic hybrids by protoplast fusion in the moss, *Physcomitrella patens*. Mol. Genet. Genom..

[26] Schneider C.A., Rasband W.S., Eliceiri K.W. (2012). NIH Image to ImageJ: 25 years of image analysis. Nat. Chem. Biol..

[27] Chiariello N.R., Mooney H.A., Williams K., Pearcy R.W., Ehleringer J.R., Mooney H.A., Rundel P.W. (1989). Growth, carbon allocation and cost of plant tissues. Plant Physiological Ecology: Field Methods and Instrumentation.

[28] Burgess J., Linstead P.J. (1981). Studies on the growth and development of protoplasts of the moss, *Physcomitrella patens*, and its control by light. Planta.

[29] Jenkins G.I., Cove D.J. (1983). Light requirements for regeneration of protoplasts o themoss *Physcomitrella patens*. Planta.

[30] Cove D.J., Quatrano R.S., Wood A.J., Oliver M.J., Cove D.J. (2004). The Use of Mosses for the Study of Cell Polarity. New Frontiers in Bryology: Physiology, Molecular Biology and Functional Genomics.

[31] Thelander M., Olsson T., Ronne H. (2005). Effect of the energy supply on filamentous growth and development in *Physcomitrella patens*. J. Exp. Bot..

[32] Bhatla S.C., Kiessling J., Reski R. (2002). Observation of polarity induction by cytochemical localization of phenylalkylamine-binding sites in regenerating protoplasts of the moss *Physcomitrella patens*. Protoplasma.

[33] Ötvös K., Pasternak T.P., Miskolczi P., Domoki M., Dorjgotov D., Szcs A., Bottka S., Dudits D., Fehér A. (2005). Nitric oxide is required for, and promotes auxin-mediated activation of, cell division and embryogenic cell formation but does not influence cell cycle progression in alfalfa cell cultures. Plant J..

[34] Prado A.M., Porterfield D.M., Feijó J.A. (2004). Nitric oxide is involved in growth regulation and re-orientation of pollen tubes. Development.

[35] Prado A.M., Colaço R., Moreno N., Silva A.C., Feijó J.A. (2008). Targeting of Pollen Tubes to Ovules Is Dependent on Nitric Oxide (NO) Signaling. Mol. Plant.

[36] He J.-M., Bai X.-L., Wang R.-B., Cao B., She X.-P. (2007). The involvement of nitric oxide in ultraviolet-B-inhibited pollen germination and tube growth of *Paulownia tomentosa* in vitro. Physiol. Plant..

[37] Šírová J., Sedlářová M., Piterková J., Luhová L., Petrivalsky M. (2011). The role of nitric oxide in the germination of plant seeds and pollen. Plant Sci..

[38] Jiménez-Quesada M.J., Carmona R., Lima-Cabello E., Traverso J.Á., Castro A.J., Claros M.G., Alché J.D.D. (2017). Generation of nitric oxide by olive (*Olea europaea* L.) pollen during in vitro germination and assessment of the S-nitroso- and nitro-proteomes by computational predictive methods. Nitric Oxide.

[39] Wang Y., Chen T., Zhang C., Hao H., Liu P., Zheng M., Baluška F., Šamaj J., Lin J. (2009). Nitric oxide modulates the influx of extracellular Ca^2+^ and actin filament organization during cell wall construction in *Pinus bungeanapollen* tubes. New Phytol..

[40] Benko P., Jee S., Kaszler N., Fehér A., Gémes K. (2020). Polyamines treatment during pollen germination and pollen tube elongation in tobacco modulate reactive oxygen species and nitric oxide homeostasis. J. Plant Physiol..

